# Exploring Mental Health and Illness in the UK Sports Coaching Workforce

**DOI:** 10.3390/ijerph17249332

**Published:** 2020-12-13

**Authors:** Andy Smith, David Haycock, Jon Jones, Kenny Greenough, Rachel Wilcock, Ian Braid

**Affiliations:** 1 Department of Sport and Physical Activity, Edge Hill University, St Helens Road, Ormskirk L39 4QP, UK; david.haycock@edgehill.ac.uk (D.H.); Greenhok@edgehill.ac.uk (K.G.); Wilcockr@edgehill.ac.uk (R.W.); 2 Thrive Approach, The Quadrangle, 1 Seale Hayne, Howton Road, Newton Abbot TQ12 6NQ, UK; Jon.Jones@thriveapproach.com; 3 DOCIAsport, Sussex Innovation Centre, University of Sussex, Science Park Square, Brighton BN1 9SB, UK; ian@dociasport.co.uk

**Keywords:** community, duty of care, mental illness, stigma, work

## Abstract

There is growing international concern about the mental health of those who work in sport, including coaches. However, we currently know little about the prevalence of mental illness and the experience of mental health among coaches, and their perceptions and use of workplace mental health support services. Little is also known about coaches’ disclosure of mental illness to, and seeking help from, work colleagues. We explore these issues using data from 202 coaches who responded to the first United Kingdom survey of mental health in the sport and physical activity workforce. In total, 55% of coaches reported having ever experienced a mental illness, and 44% currently did, with coaches in grassroots/community settings being most likely to experience mental illness. Depression and anxiety were the most commonly reported conditions and many coaches preferred to access mental health support outside of the organisation for whom they worked or volunteered, with decisions to seek help from others in the workplace being shaped by complex organisational and personal considerations. The findings suggest there is an important public health challenge which needs to be met among coaches, so that we can better address a question of fundamental importance: ‘who is looking after the people looking after the people’?

## 1. Introduction

There is growing international concern about individual and population mental health, and the prevalence of mental illness, whether in sport [1,2] or other aspects of the wider society [3]. (Appendix A) In a sporting context, increasing attention has been paid to the mental health and illness of various groups, including participants, coaches and officials in community (local level) and high-performance sport (at national and international levels). Studies of sports coaches’ mental health have typically been derived from psychology (especially organisational psychology), and have focused on, among other things, coaches’ perceptions and experiences of workplace stressors [4,5,6,7,8], work–life balance, stress, coping and burnout [9,10,11,12], mental health literacy [13], job control, insecurity and mental wellbeing [5,9], and mental health awareness and seeking help from others [14,15]. Other research has focused on the need to explore other mental health parameters than burnout in coaches [16], the role coaches are expected to play in supporting the mental health of others, especially young people [17,18], parents’ views on the roles of coaches in supporting mental health [19,20], and the need to provide coaches with evidence-based guidance on mental health to help facilitate the inclusion of participants who experience, or have experienced, mental illness [21]. Several international consensus statements [1,22,23,24] have pointed to the important role coaches are expected to play in supporting athlete mental health, seeking help for their own mental health, the need for coach education and training programmes focused on mental health and suicide, and the role of coaches (and others) in co-creating mental health promotion in diverse sport settings.

Although much less common and developed [2,25,26], the existing sociological studies of sport have examined—often indirectly—some keys aspects of sports coaches’ mental health. These include the impact of enacting government community sport policy on wellbeing [27,28,29], the centrality of emotions and social relations to coaching practice [30,31,32], the use of self-coping strategies such as alcohol consumption to manage experiences of depression [33], notions of care and caring practices in coaching work [34,35], and the management of personal lives with occupational demands in female high-performance sports coaches [36].

In the United Kingdom (UK), coach mental health, the role of coaches in supporting others’ mental health, and the responsibilities of organisations in supporting the mental health of coaches, were recently considered as part of the mental welfare theme of an independent review of duty of care in sport led by Baroness Grey-Thompson, and involving two of the authors of this article (A.S., I.B.), in 2017 [37]. Among the priority recommendations was the suggestion that the UK government should create a Sports Ombudsman which ‘should have powers to hold national governing bodies [NGBs] to account for the Duty of Care they provide to all athletes, coaching staff and support staff, providing independent assurance and accountability to address many of the issues covered by this review’ [37] (p. 6). A Duty of Care Charter was also identified as an important step through which the government should articulate how ‘participants, coaches and support staff can expect to be treated and where they can go if they need advice, support and guidance’ [37] (p. 6). In relation to mental health, four specific recommendations were also made: (i) ‘introduce sector standard mental health training for coaches and physical activity professionals (which can have aspects of sports specificity where required)’; (ii) ‘NGBs to include mental health issues in the content of coaching and other sport-related courses which filter down to clubs’; (iii) ‘staff, coaches and athletes to receive mental health awareness training and support, which should be included as part of induction processes as well’; and (iv) ‘NGBs and sports clubs should provide clear signposting to confidential support services outside of the sport (for participants, coaches and performance staff)’ [37] (p. 22). At the time of writing, none of these recommendations have been formally implemented by the government, but progress has been made in relation to them by several organisations. These include UK Coaching who, in partnership with the national mental health charity Mind, as well as Sport England and 1st4Sport, have developed online mental health awareness for sport and physical activity training for coaches, and identified mental health as one of five pillars of its Duty to Care Toolkit and Digital Badge [38]. It is too early to determine the effectiveness of these developments, but they nevertheless build upon the increased attention which has come to be placed upon the relationship between work and mental health following the proposed introduction of new mental health standards in workplaces [39], including in relation to employee mental health and wellbeing in sport and physical activity [40].

Recognising the importance of mental health in coaching, including coaches’ mental health and the mental health of the coach developers who educate coaches, is important given the increased alleged political and policy commitment to addressing mental health, illness and wellbeing [29,41] in and through coaching across all levels of the sport and physical activity landscape [38,42,43,44], and the significant costs of mental illness to individuals, communities and whole societies. Despite the progress which has been made in the research, policy and practice related to coaching and mental health, we currently know little about the ‘state of play’ in this area, and especially the prevalence of mental illness and experiences of mental health among coaches, their perceptions and their use of workplace mental health support services, and coaches’ disclosure of mental illness to, and seeking of help from, others with whom they work. To address this significant gap in understanding, the aim of this paper is to present new empirical data on the mental health of UK coaches, and their experience of workplace mental health, across all levels of sport (i.e., grassroots/community sport, high-performance sport, activity/lifestyle/recreation and education). In so doing, we draw upon coaches’ responses to what is to our knowledge the first UK survey of mental health in the sport and physical activity workforce, which encompasses sports coaches who are engaged in voluntary and paid (non) professional labour markets in sport, as explained next.

## 2. Materials and Methods 

### 2.1. Recruitment and Sample

Participants (aged 16 and above) were recruited by personal and wider organisational networks across the UK as part of a cross-sectional study. The sample of coaches was derived from 1201 respondents who at the time of being surveyed were employed (or contracted) in a paid position, or acted as a volunteer/on a voluntary basis for any organisation in the sport and physical activity workforce. The organisations by whom coaches were employed were deliberately diverse so as to attract as many respondents as possible, and included those coaches who worked for the following: charities and voluntary groups; coaching providers; community interest companies; education (e.g., schools, colleges, universities); exercise, physical activity and leisure providers; government-funded departments; national governing bodies; non-departmental public bodies; outdoor education and activity providers; professional bodies/organisations; professional player associations; professional teams or squads; sports clubs; and sport-for-development/social change organisations. Overall, 202 coaches working in over 30 sports or physical activities, at the grassroots/community level (e.g., NGBs, sports clubs, teams), in high-performance sport (e.g., Olympic and Paralympic sports), and in the activity/lifestyle/recreation (e.g., private gyms, running, cycling) and education sectors (e.g., further education colleges and universities), were included in the wider sample and their responses are examined in this article.

### 2.2. Instrument

An online survey instrument (the Sport and Physical Activity Workforce Mental Health (SPAWMH) Survey) was used to generate demographic data on respondents’ backgrounds (e.g., age, sex, gender, sexuality, disability, ethnicity, sector of employment, sport/activity coached) and their experience of mental health (including whether they had personally ever experienced, or currently are experiencing, mental illness, type of mental illness experienced, mental health awareness). The respondents’ perceptions and experiences of mental health support and seeking help (including workplace mental health disclosures, engagement in mental health services and organisational policies related to mental health), were also examined. The survey questions were developed from insight derived from existing resources on workplace mental health [39,40], and internationally used categories of mental illness or disorder [3].

Responses were captured using a range of Likert-type scales and closed single-/multi-answer questions which generated quantitative data, while free-text, open-ended questions were used to capture participants’ qualitative experiences of the issues covered. These open text responses provided opportunities for respondents to convey their understanding of the terms ‘mental health’ and ‘mental illness’, rather than providing them with formal definitions of these. Other areas on which respondents were able to provide open text responses included their perceptions and experiences of mental health service use, reasons for disclosing mental illness (or not) to others in the workplace, and perceptions of the seriousness with which mental health and illness are taken by their leader(s) and manager(s).

### 2.3. Procedure and Ethical Approval

Participants completed the SPAWMH survey using the Online Surveys software (Bristol, UK) between 10 October (Mental Health Awareness Day) and 31 December 2018. Unless participants decided to provide personal contact information at the end of the survey to indicate their willingness to engage in a follow-up one-to-one interview, all surveys were completed anonymously. Ethical approval was provided by one of the lead author’s institutional ethics committees (SPA-REC-2018-022) and informed consent was provided by participants through the completion and submission of their survey responses.

### 2.4. Analysis

Select categorical responses were recoded into ordered categories. Age was recoded into five categories (years; 16–24, 25–34, 35–44, 45–59; >60). Confidence in speaking to others about one’s mental health was recoded into three categories (very confident or confident, neither confident nor unconfident, very unconfident or unconfident), while the likelihood of disclosing an experience of mental illness to others in the coach’s own organisation was recoded into four categories (very likely or likely, somewhat likely, very unlikely or unlikely, don’t know). The seriousness with which mental health and illness are taken by the leader(s) or manager(s) of the coach’s organisation was also recoded into three categories (not at all or not very seriously, somewhat seriously, seriously or very seriously).

Cross-tabulation and descriptive statistics (frequency counts, proportions) were used to help analyse the survey demographic information and participants’ answers to separate items in each of the four sections of the survey. For analysis purposes, where appropriate, we organised all respondents into four groups based on the sector in which they worked (i.e., grassroots/community sport, high-performance sport, activity/lifestyle/recreation and education), and whether they had ever experienced and/or were currently experiencing mental illness. Having analysed the quantitative data, we extracted all qualitative free-text responses to the relevant open-ended questions and reviewed these alongside the closed-ended questions to which they related as part of a QUANT --> qual like research design. The qualitative responses were thus used to help interpret the quantitative results, and illustrative verbatim examples are thus summarised, and integrated, here alongside the corresponding quantitative data. 

## 3. Results

Of the 202 coaches who responded to the survey, 58% were male (*n* = 118) and 42% female (*n* = 84). As Table 1 indicates, the majority were self-identified as heterosexual, non-disabled and White. In total, 47% were aged 45 and above, 20% were aged 25–34 and 24% were 35–44 years-old; 53% worked at grassroots/community level and approximately twice as many coaches worked in high-performance sport (17%) and activity/lifestyle/recreation (20%) compared to education (9%).

### 3.1. Coaches’ Experience of Mental Illness

In total, 55% of coaches had ever experienced mental illness (63% of whom had been formally diagnosed by a GP or medical professional), with higher proportions of those working at grassroots/community level and in the activity/lifestyle/recreation sector reporting this. More female coaches reported having ever experienced mental illness (65% had been formally diagnosed, as had 62% of males), with 69% of women who worked in grassroots/community sport and physical activity indicating they had done so. In total, 47% of males who worked at the grassroots/community level also reported having ever experienced mental illness. Of those coaches who had ever experienced mental illness, 44% reported they were currently experiencing it (of these, 59% had been formally diagnosed by a GP or medical professional). However, more males reported currently experiencing a mental illness such as depression or anxiety (43% had been formally diagnosed, as had 39% of females), while higher proportions of males and females who worked at the grassroots/community level reported currently experiencing a mental illness compared to those working in other sectors (Table 2). When analysed by age, higher proportions of young female coaches (aged 16–24 and 25–34) tended to report having ever experienced, or that they were currently experiencing, mental illness, while more males aged 35 and over were currently experiencing mental illness (Table 3).

Depression and anxiety were the most commonly reported conditions ever and currently experienced by coaches, with self-harm also being reported by one-fifth of females. Other, much less commonly reported, conditions which were ever experienced by coaches included panic disorders and post-traumatic stress disorder. These conditions, together with obsessive compulsive disorder and substance use disorders (including alcohol), were also reported by a very small proportion of coaches who were currently experiencing mental illness (Table 4).

### 3.2. Preferred Workplace Mental Health Support and Organisational Culture

In total, 54% of coaches (63% female, 47% male) in our sample preferred to receive mental health support exclusively from someone outside the organisation for which they worked or volunteered, and one-fifth (15% female, 26% male) preferred to receive support from someone inside and outside their organisation, while 7% of males and females preferred to receive support from someone inside their organisation. Of the coaches, 17% (14% female, 11% males) did not know whom they wished to receive mental health support from. The types of mental health support coaches would like from their organisation are presented in Table 5. Reflecting many coaches’ preference for seeking mental health support from outside their organisation, one-third (34%) of respondents would like their organisation to signpost to appropriate external services, with the next most commonly requested forms of support (for males and females) being counselling, having a healthy work-life balance and having access to regular health checks.

Eight in ten coaches (78% male, 80% female) were also unaware of any strategies or policies regarding mental health which were provided by their organisation, or any mental health support available to them in their organisation (80% male, 77% female). That being said, 48% of females and 43% of males felt that the leader(s) or manager(s) of their organisation took mental health and illness seriously or very seriously, four in ten male coaches (39%) and three in ten female coaches (29%) felt they did so somewhat seriously, and one-quarter (22% male, 24% female) of coaches said the leader(s) or manager(s) of their organisation did not take mental health and illness at all seriously, or took it not very seriously. The responses given in relation to the seriousness with which mental health and illness were perceived to be taken by leader(s) or manager(s) are presented in Table 6, which provides examples of the verbatim free-text comments provided by coaches and details on the sports/physical activities in which they worked. The comments demonstrate clearly the significant variability in how mental health and illness are perceived and understood in the workforce, and how seriously they are likely to be treated and supported by managers and organisations.

### 3.3. Mental Illness Disclosure and Seeking Help at Work

Two-thirds of males (65%) and 53% of females suggested that it was very unlikely or unlikely that coaches would disclose experience of mental illness to others in their organisation, while one-third (33%) of female coaches and one-fifth (21%) of male coaches felt it was somewhat likely. Very few coaches felt it was very likely or likely (11% females, 5% males) that coaches would disclose an experience of mental illness to others in their organisation and 6% did not know (4% female, 6% male).

Three in ten coaches (29% females, 30% males) suggested that they felt unconfident or very unconfident speaking to others in their organisation about their mental health, while 47% of males and 46% of females reported feeling confident or very confident doing so. One-quarter (24%) indicated they were neither confident nor unconfident (25% female, 23% male) of speaking to others in their organisation about their mental health. In addition, one-quarter of coaches (24% male, 27% female) had spoken to others in their organisation about their mental health, 13% (14% male, 12% female) had not spoken to others but would have liked to, and 62% (63% male, 61% female) had not spoken to others and had not needed to. Those coaches who indicated they had not spoken to others about their mental health, but would have liked the opportunity to do so, were also given the opportunity to include free-text comments to explain why. Examples of the responses provided are included in Table 7, which indicates the significance of perceptions of shame, weakness, and other negative perceptions of mental health among coaches who often occupy highly pressurised roles. As leaders of others, the comments also indicate how coaches have a perceived responsibility to portray stoicism, to not show weakness, and appear in control of themselves and the situations with which they are faced as competent, confident and strong people. 

In total, 58% of males and females had not received nor required support for their mental health from someone in their organisation, and 28% had not asked for support (29% males, 27% females), but 12% of males and females had received support for their mental health from someone in their organisation. Just 2% of male and female coaches had sought support from others but had not received that support. Examples of the varied responses coaches gave when asked to explain whether they had received support for their mental health from others in their organisation are provided in Table 8. For some coaches, it was clear that they received important support from others inside their organisation (e.g., Safeguarding Officer, counsellors, other coaches and psychologists), while for others they did not receive the support they needed (e.g., because of a lack of understanding by others, or failure by employers to deal with sources of anxiety), or did not wish to receive support (e.g., because mental health was perceived as a personal responsibility, or support was received from family).

## 4. Discussion

The purpose of this paper has been to present new empirical data on the prevalence and experience of mental health and illness among UK sports coaches, their use of workplace mental health support services, and coaches’ disclosure of mental illness and seeking help from others with whom they work. Overall, 55% of coaches reported ever experiencing a mental illness, and 44% of coaches were experiencing a mental illness at the time of being surveyed. Although not directly comparable, there were more than twice as many coaches in this study who reported ever experiencing a mental illness compared to one-fifth (19.4%) of UK coaches who reported a previous diagnosis of a mental disorder in Gorczynski et al.’s study [13], and the proportion of those who were currently experiencing mental illness was slightly lower than those coaches (49.5%) who were exhibiting symptoms of a mental disorder at the time Gorczynski et al. [13] conducted their investigation. Depression and anxiety were the two most commonly reported mental illnesses by coaches in the present study, which was perhaps unsurprising given that depression and anxiety disorders are the most prevalent mental illnesses recorded globally, including in the UK [3].

The findings also revealed that an experience of mental illness (ever and currently) was most commonly reported by coaches working or volunteering at the grassroots/community level. Although our sample included disproportionately more coaches working in local communities, this is where the majority of coaches in the UK (as in many other countries) operate, followed by those working in related areas, such as the activity/lifestyle/recreation and education sectors [43,44]. Thus, an important public mental health challenge in sports coaching appears to be among those coaches working in grassroots/community sport and physical activity. This is not to suggest that the mental health and illness of high-performance professionals is unimportant, since previous research has demonstrated convincingly how high-performance coaches experience various personal, performance and organisational stressors which are related to high-performance sports cultures that often impact the mental health of coaches [4,5,6,13]. These include the following: the tendency for the success and continued employment of high-performance coaches to be largely dependent upon athlete performance; many high-performance coaches are also self-employed and retained on short-term contracts; and in Olympic and Paralympic sports especially, coaches’ employment and mental health are typically tied to the constraints of four-yearly funding cycles. However, it is clear that there is also a significant need to support the mental health of grassroots/community coaches, including through mental health awareness training and support, and interventions which address the diverse sources of key stressors inside or outside of their organisation, including the constraints associated with balancing other work and family commitments and negotiating the pressures exerted from significant others [1,5,13,21,37]. Our findings suggest that, for many coaches, services provided by people outside of the organisations were most preferred, while those provided exclusively inside the organisation for which they work, or volunteer, were least preferable. This is consistent with the findings of Gorczynski et al. [13], who noted how the coaches in their sample preferred to seek support for their poor mental health from partners, family and friends rather than others outside the workplace, including mental health professionals and their general practitioners.

For Gorczynski et al. [13], coaches’ lack of willingness to engage with mental health professionals and associated services may be related to poor service availability and access, limited mental health literacy, and general intentions to seek help from others. In the present study, coaches’ willingness to engage, or not, in seeking help inside their workplace was shaped by their relations with others (especially colleagues and managers), traditional perceptions of mental illness as a source of stigma, shame, fear, weakness and embarrassment in sport and other aspects of the wider society [2,45], and concerns about job security. Fear of losing one’s job [5,9], of having one’s credibility and ability to fulfil role expectations questioned, and of risking disciplinary action were indeed of particular concern to coaches in our sample, and were consistent with the experiences of other employees [39,40]. Conversely, positive workplace relations based on trust, confidentiality and a concern with setting an example to others were identified as important reasons for seeking support from others in the workplace [39,40]. The kind and quality of coaches’ workplace relations, an ability to achieve a healthy work–life balance and high levels of workplace control [5,9,26], the effective management of boundaries between work and other aspects of life [26,36], and the avoidance of role ambiguity and having too many responsibilities are thus among the effective ways of safeguarding personal mental health and maximising performance [5]. Together with high levels of mental health literacy and supportive workplace mental health cultures [13], they also provide the foundations upon which coaches might effectively support the mental health of others, which is one among many responsibilities coaches now assume across all levels of sport and physical activity [16,17,18,21], and in many countries [1,22,23,24].

There are several strengths and limitations in this study. By drawing upon findings from the UK’s first survey of mental health in the sport and physical activity workforce, this study has provided a novel preliminary insight into the prevalence and disclosure of mental illness among coaches, their use of workplace mental health services and their engagement in seeking help from others. It has drawn new attention to the mental health of grassroots/community coaches, in particular, and those in the activity/lifestyle/recreation and education sectors, which have been largely under-researched in comparison to coaches who work in high-performance sport. However, much research still needs to be done to better understand the mental health of high-performance sport coaches. The findings demonstrate the public health challenge of supporting mental health and illness among community-based coaches, and in so doing identify the service use and other support matters which need to be considered as part of that public health effort. These are also challenges faced by organisations across the UK sport and physical activity landscape, and our findings provide a useful starting point for informing the design and delivery of coach education and development programmes, and associated duty of care practices, which need to take greater account of the mental health needs of coaches (including coach developers and coach educators) and the participants for whom they are responsible, and the various workplace and sports cultures in which they exist. No less importantly, our findings also point to the importance of creating a better, more mentally healthy, working environment across all sports, and all levels of sport, to attract and retain—on an equitable and diverse basis—the next generation of coaches who will be critical for the future sustainable delivery of sport and physical activity. 

This having been said, it is important to remember that our data are derived from a cross-sectional study based on self-report data, so we are unable to establish any form of causation–only relationships, and should remain mindful of the potential of social desirability bias in coaches’ responses. As is common in other forms of survey research, there is also the possibility of survival bias, since we recruited only currently working or volunteer coaches, and not those who have previously worked in this area. Self-selection bias was also possible, and it is important that future studies seek to address this in their designs in this considerably under-researched area. Finally, we do not claim that the findings are representative of all coaches, working across similar sectors, in the UK, though it is important future investigations seek to better understand the mental health of different groups of coaches (and their intersecting identity characteristics), working in different organisational contexts, across different regions and settings. Future research also needs to examine, among other things, the following in greater detail: the prevalence and patterns of mental illness among coaches; how coaches manage their own and others’ mental health, including through seeking help from others and service use [4]; the mental health literacy of coaches and how this can be developed [13]; the additional resources and support needed to help coaches (in community and high-performance settings) engage in their expanded role related to mental health promotion [38,43,44]; how the enhancement of mental health, as an aspect of wider duty of care activity [34,35,37,38], might be associated with improved outcomes for coaches, participants and organisations; and how coaches’ embodied experiences of mental health and illness shape their lives inside and outside of the workplace [2,26].

## 5. Conclusions

This study has offered novel insight into the mental health of coaches, working in a paid or voluntary capacity, across the UK sport and physical activity landscape. It has demonstrated how mental illness was previously experienced by 55% of coaches, and 44% were experiencing a mental illness at the time the study was conducted. Our data indicate, however, that the prevalence of mental illness varies between the workplace sectors in which coaches were based, with those coaching in grassroots/community settings being the most likely to report experiencing mental illness. Coaches’ preferences for engaging with mental health support services outside their organisation, and decisions about whether to seek support from others, have important policy and practice implications in sport and public health terms. In particular, the study highlights the need to pay greater attention to mental health in the sector where most coaches are employed or volunteer, namely, grassroots/community settings, while retaining an equally important focus on the mental health needs of those who work in high-performance sport. The study also makes clear how coaches’ experiences of mental health and illness, and their use of relevant support services and engagement in seeking help from others, are contextually and situationally contingent. They need, moreover, to be understood in the context of coaches’ workplace relations, practices and experiences, and their interdependence with other aspects of coaches’ personal lives and the wider political and policy environments in which they enact their work. Overall, our data indicate clearly the need to pay greater attention to, and take seriously, the mental health needs of coaches so that we are in a much better position to address a question of fundamental importance, emphasised repeatedly by the last author: ‘Who is looking after the people looking after the people’?

## Figures and Tables

**Table 1:** Demographic characteristics of sample (*n* and %).

Variable	Total (*n* = 202)	Males (*n* = 118)	Females (*n* = 84)
Age			
16–24	17 (8.4)	7 (5.9)	10 (11.9)
25–34	41 (20.3)	23 (19.5)	18 (21.4)
35–44	49 (24.3)	23 (19.5)	26 (31.0)
45–59	73 (36.1)	47 (39.8)	26 (31.0)
60+	22 (10.9)	18 (15.3)	4 (4.7)
Sexuality			
Heterosexual	189 (93.6)	115 (97.5)	74 (88.1)
Gay man	1 (0.5)	1 (0.8)	0 (0.0)
Gay woman/lesbian	4 (2.0)	0 (0.0)	4 (4.8)
Bisexual	5 (2.5)	1 (0.8)	4 (4.8)
Prefer not to say	3 (1.4)	1 (0.8)	2 (2.4)
Ethnicity			
White British	134 (66.3)	76 (64.4)	58 (69.0)
White English	31 (15.3)	21 (17.8)	10 (11.9)
White Irish	11 (5.4)	7 (5.9)	4 (4.8)
White Scottish	10 (5.0)	3 (2.5)	7 (8.3)
White Welsh	6 (3.0)	5 (4.2)	1 (1.2)
Other/prefer not to say	10 (5.0)	5 (4.2)	4 (4.8)
Disabled	11 (5.4)	5 (4.2)	6 (7.1)
Physical disability	3 (1.5)	2 (1.7)	1 (1.2)
Learning disability	6 (3.0)	3 (1.5)	3 (3.6)
Hearing impairment	2 (1.0)	0 (0.0)	2 (2.4)
Prefer not to say	1 (0.5)	1 (0.8)	0 (0.0)
Sector worked			
High-performance sport	35 (17.3)	25 (21.1)	10 (11.9)
Grassroots/community	108 (53.4)	56 (47.5)	52 (61.9)
Education	19 (9.4)	12 (10.2)	7 (8.3)
Activity/lifestyle/recreation	40 (19.8)	25 (21.2)	15 (17.9)

**Table 2:** Workforce sector-related experience of mental illness (*n* and %).

Workforce Sector	Total (*n* = 202)	Males (*n* = 118)	Females (*n* = 84)
Ever experienced mental illness	111 (55.0)	60 (50.8)	51 (59.5)
High-performance sport	16 (14.4)	11 (18.3)	5 (9.8)
Grassroots/community	63 (56.8)	28 (46.7)	35 (68.6)
Education	10 (9.0)	7 (11.7)	3 (5.9)
Activity/lifestyle/recreation	22 (19.8)	14 (23.2)	8 (15.7)
Currently experience mental illness ^1^	49 (44.1)	28 (46.7)	21 (41.2)
High-performance sport	6 (12.2)	3 (10.7)	3 (14.3)
Grassroots/community	33 (67.3)	17 (60.7)	16 (76.2)
Education	2 (4.1)	2 (7.1)	0 (0.0)
Activity/lifestyle/recreation	8 (16.3)	5 (17.9)	3 (14.3)

^1^ Of those who ever experienced mental illness.

**Table 3:** Age-related experience of mental illness (*n* and %).

Age Group	Total	Ever Experienced Mental Illness	Currently Experiencing Mental Illness ^1^
Males	118 (100.0)	60 (50.8)	28 (46.7)
16–24	7 (5.9)	5 (71.4)	3 (60.0)
25–34	23 (19.5)	11 (47.8)	6 (54.5)
35–44	23 (19.5)	12 (52.2)	7 (58.3)
45–59	47 (39.8)	23 (48.9)	11 (47.8)
60+	18 (15.3)	8 (44.4)	0 (0.0)
Females	84 (100.0)	51 (59.5)	21 (41.2)
16–24	10 (11.9)	7 (72.7)	4 (57.1)
25–34	18 (21.4)	13 (72.2)	9 (69.2)
35–44	26 (31.0)	17 (65.4)	6 (35.3)
45–59	26 (31.0)	10 (38.5)	1 (10.0)
60+	4 (4.8)	3 (75.0)	1 (33.3)

^1^ Of those who ever experienced mental illness.

**Table 4:** Top five mental illness conditions experienced by coaches (*n* and %).

Mental Illness	Total (*n* = 202)	Males (*n* = 118)	Females (*n* = 84)
Ever experienced	111 (55.0)	60 (50.8)	51 (59.5)
Depression	80 (72.1)	44 (73.3)	36 (70.6)
Anxiety	72 (64.9)	42 (70.0)	30 (58.8)
Panic disorders	18 (16.2)	10 (16.7)	8 (15.7)
Self-harm	17 (15.3)	6 (10.0)	11 (21.6)
Post-traumatic stress disorder	16 (14.4)	8 (13.3)	8 (15.7)
Currently experienced ^1^	49 (44.1)	28 (46.7)	21 (41.2)
Anxiety	31 (63.3)	20 (71.4)	11 (52.4)
Depression	29 (59.2)	19 (67.9)	10 (47.6)
Self-harm	7 (14.3)	3 (10.7)	4 (19.0)
Obsessive compulsive disorder	4 (8.2)	3 (10.7)	1 (4.8)
Post-traumatic stress disorder	3 (6.1)	1 (3.6)	2 (9.5)
Substance use disorder (including alcohol)	3 (6.1)	3 (10.7)	0 (0.0)

^1^ Of those who ever experienced mental illness.

**Table 5:** Type of mental health support coaches would like from their organisation (*n* and %).

Method of Support	Total (*n* = 202)	Males (*n* = 118)	Females (*n* = 84)
Signposting to Appropriate External Services	69 (34.2)	37 (31.4)	32 (38.0)
Counselling	64 (31.7)	40 (33.9)	24 (28.6)
Healthy Work–Life Balance	56 (27.7)	36 (30.5)	20 (23.8)
Regular Health Checks	42 (20.8)	25 (21.2)	17 (20.2)
Cognitive Behavioural Therapy	36 (17.8)	12 (10.2)	14 (16.7)
Flexible Working	30 (14.9)	13 (11.0)	17 (20.2)
Psychologist	30 (14.9)	18 (15.3)	12 (14.3)
Occupational Health/Employee Assistance Programme	27 (13.4)	14 (11.9)	13 (15.5)
Digital/Online Support	12 (5.9)	2 (1.7)	10 (11.9)

**Table 6:** Coaches’ perceptions of the seriousness with which mental health and illness are taken by their leader(s) and manager(s).

Males	Females
Football: ‘I can only talk from my own experiences, but the support I have received has been top class. The best thing I have done this year is disclose my mental illness to the club and I feel it will have a profound impact on my life moving forward.’	Gymnastics: ‘Difficult to say for sure-mental health has literally never been discussed in the team or by the manager of our organisation’.
Rugby league: ‘If you had any issues the bosses would in 99% of cases wouldn’t be interested.’	Football: ‘They make people very aware that it’s okay to not be okay, to ask for support.’
Futsal: ‘I believe things are improving in areas of our sector but there is still a lot of work to do for leaders and organisations to truly understand the impact and implications on their employees and the steps and processes that should be in place to support people.’	Triathlon: ‘I do not know where staff can go and if they wish to acknowledge issues as, unfortunately, they may still think they have to be strong for the teams/athletes they work with. We are recognising MH but still need to find solutions and promote the success of them to share that poor MH is common and ok.’
Running: ‘There is very little understanding of mental illness or well-being. I think it can be perceived as trouble making.’	Swimming: ‘If discussed to someone in my organisation I believe the seriousness would be dependent on whom it was disclosed to.’
Triathlon: ‘I think it is more and more something that is thought about but still not a priority.’	Hockey: ‘As a volunteer organisation we don’t even consider the mental health of the volunteers in roles.’
Multi-sport: ‘In my sector, mental health needs to be taken very seriously however the way it is dealt with may not be what is required as people often mistake it for something that is dangerous.’	Canoeing: ‘I don’t think the parents of children in the club I volunteer for consider the mental wellbeing of the volunteers and perhaps it would be helpful if they could be educated on the needs of the club as a whole and the volunteers within it. I don’t hold the club committee responsible, I just think it’s something we could look at and put something in place to help members understand the effect their behaviours may have on the volunteers within a club.’
Athletics: ‘If in a leadership role or/and a competitive environment, I know it can be used to discredit me.’	Multi-sport: ‘we are supported by all managers. There have been cases that we are aware of that have been dealt with appropriately and made a priority.’

**Table 7:** Why coaches did not speak to others about their mental health, despite wanting to.

Males	Females
Hockey: ‘In the position of coach, it’s incredibly high pressure. I didn’t feel I was able to discuss my mental health with anyone, as I didn’t want to appear weak in the role.’	Boxing: ‘Shame, think they would tell others.’
Gymnastics: ‘Because I hear the gossip and negative small talk around such subjects and the manner in which they use that knowledge to further make work life uncomfortable.’	Squash: ‘I’ve always seen it as a negative that I was struggling, and didn’t want people to think I was failing at something.’
Cricket: ‘I was stressed because I’m at university, but I also work for a couple of professional sports teams and volunteer at my home club. This resulted in a lot of work without much time, leading to stress and feeling unable to cope, however I felt as though I couldn’t say anything or ask for less work as it would be a sign of being unable to cope, therefore not suitable for a potential future job.’	Running: ‘As the coach it’s sometimes difficult to be ‘real’? I’m supposed to be the strong one of my team of leaders!’
Goalball: ‘Because I don’t want to be a burden on others and often I am the person in charge of the program and supporting others who are struggling so I feel I need to portray a strong character who is always happy/confident and coping well.’	Swimming: ‘Those in a position of responsibility i.e., The committee are unresponsive and do not seem to be trained to cope with such disclosures. The welfare officer is but rarely present.’
Multi-sport: ‘I feel if I speak up about my health it would effect (sic) my work e.g., lose my job, be forced into time off etc.’	Gymnastics: ‘Worrying about being seen as incompetent, especially where I am responsible for safeguarding children.’
Athletics: ‘Don’t know who will listen, how they will judge you.’	Multi-sport: ‘Fear of disciplinary procedures, fear of judgement or perceived incapability. Mental illness shouldn’t define you but once a disclosure is given there is often concern around your abilities.’

**Table 8:** Coaches’ responses to whether they had received support for their mental health from others in their organisation.

Males	Females
Football: ‘I had a professional contract. I was released at 19 years of age. I struggled for many years with my identity. I often identified myself as a failed footballer and couldn’t see a future. Living like this led to issues with alcohol and gambling…For many years I felt like I didn’t want to carry on with my life. I carried on doing things, surviving as such…I reached out to the Safeguarding Officer at our club and he directed me to the PFA who sorted…a local psychotherapist. I feel this support has helped me come to terms with my own life…I [now] feel proud of what I do now and see a future for myself.’	Football: ‘I struggled to cope with the pressures of being a new female coach juggling lots of other activities at work and home life on top of managing a team. I [wasn’t] introduced to most other people from the club so couldn’t have spoken to them.’
Cricket: ‘I’ve never asked. However, when I was 16, I felt really stressed/maybe a bit depressed and I rang up my local mental health number and I was forwarded to somewhere else because I wasn’t in the right postcode, the next number then gave me another number and then they gave me another. In the end I gave up, when all I needed was someone to talk to. Also, [I] wouldn’t know where to go now if I felt stressed as an adult male.’	Running: ‘There are healthy friendships within the club. I am seen as approachable and able to maintain confidentiality [and] have health care experience.’
Basketball: ‘Members from the organization have helped me to withstand stressful situations and allowed me to continue working normally.’	Archery: ‘Maintaining my club membership and participation in my sport was one of the factors in my recovery from my own mental health issue. It provided a space in which I could still engage with all that was positive and uplifting in my life.’
Hockey: ‘I went directly to counselling and kept it quiet, I did not want to speak to others.’	Athletics: ‘I needed support to tackle the person causing my anxiety/stress but nothing was done.’
Athletics: ‘At present I don’t feel I need to speak to them about it as I receive a lot of support from my wife, also I would not want them to judge my ability to carry out my role.’	Squash: ‘I didn’t feel I could ask for help.’
Triathlon: ‘I have some very close working relationships with other coaches and also our team’s ex-psychologist. I felt in my role as head coach, that I have a responsibility to be open about how I feel about things to help ensure those around me feel comfortable to do the same.’	Diving: ‘I feel that my health problems are mine and mine alone. Other people do not have the patience or time, and many do not understand.’
American Football: ‘I am an unpaid volunteer coach. I use my coaching role as part of my recovery plan. If I need to bow out of any activities, I can do so without fear of ‘persecution’.’	Running: ‘I was aware of what I needed to do and got help externally. This was enough for me.’
Running: ‘I have always been concerned with how other people will view me when it comes to my past and believed that this would have [a] negative impact [on] the trust relationship you build with your athletes as a coach.’	Netball: ‘I am leaving my current position through the lack of support on mental health issues.’

## Appendix A

In this paper we regard mental health as ‘a state of well-being in which every individual realizes his or her own potential, can cope with the normal stresses of life, can work productively and fruitfully, and is able to make a contribution to her or his community’ (p. 12) [46]. In contrast, mental illness (also referred to as mental or psychiatric disorders) is any health condition (such as depression and anxiety) which involves changes or alterations in thinking, mood or behaviour (or any combination of these) which can result from stressful experiences, but also occur in the absence of such experiences [46].

## References

[1] Breslin G., Smith A., Donohue B., Donnelly P., Shannon S., Haughey T., Vella S., Swann C., Cotterill S., MacIntyre T. (2019). International Consensus statement on psychosocial and policy-related approaches to mental health awareness programmes in sport. BMJ Sport Exerc. Med..

[2] Smith A., Atkinson M. (2019). Depression and suicide in professional sports work. Sport, Mental Illness and Sociology.

[3] World Health Organization (2017). Depression and other Common Mental Disorders: Global Health Estimates.

[4] Bissett J., Kroshus E., Hebard S. (2020). Determining the role of sport coaches in promoting athlete mental health: A narrative review and Delphi approach. BMJ Open Sport Exerc. Med..

[5] Carson F., Malakellis M., Walsh J., Main L.C., Kremer P. (2019). Examining the mental well-being of Australian sport coaches. Int. J. Environ. Res. Public Health.

[6] Fletcher D., Scott M. (2010). Psychological stress in sports coaches: A review of concepts, research, and practice. J. Sports Sci..

[7] Norris L., Didymus F., Kaiseler M. (2017). Stressors, coping, and well-being among sports coaches: A systematic review. Psychol. Sport Exerc..

[8] Thelwell R., Wagstaff C., Chapman M., Kenttä G. (2017). Examining coaches’ perceptions of how their stress influences the coach–athlete relationship. J. Sports Sci..

[9] Bentzen M., Kenttä G., Richter A., Lemyre P.-N. (2020). Impact of job insecurity on psychological well- and ill-being among high performance coaches. Int. J. Environ. Res. Public Health.

[10] Bentzen M., Lemyre P.-N., Kenttä G. (2016). Changes in motivation and burnout indices in high-performance coaches over the course of a competitive season. J. Appl. Sport Psychol..

[11] Didymus F. (2017). Olympic and international level sports coaches’ experiences of stressors, appraisals, and coping. Qual. Res. Sport Exerc. Health.

[12] Olusoga P., Butt J., Hays K., Maynard I. (2009). Stress in elite sports coaching: Identifying stressors. J. Appl. Sport Psychol..

[13] Gorczynski P., Gibson K., Clarke N., Mensah T., Summers R. (2020). Examining mental health literacy, help seeking behaviours, distress, and wellbeing in UK coaches. Eur. Phys. Ed. Rev..

[14] Breslin G., Shannon S., Haughey T., Donnelly P., Leavey G. (2017). A systematic review of interventions to increase awareness of mental health and well-being in athletes, coaches and officials. Syst. Rev..

[15] Hegarty E., Weight E., Register-Mihalik J. (2018). Who is coaching the coach? Knowledge of depression and attitudes toward continuing education in coaches. BMJ Open Sport Exerc. Med..

[16] Olusoga P., Bentzen M., Kenttä G. (2019). Coach burnout: A scoping review. Int. Sport Coach. J..

[17] Ferguson H., Swann C., Liddle S., Vella S. (2019). Investigating youth sports coaches’ perceptions of their role in adolescent mental health. J. Appl. Sport. Psychol..

[18] Mazzer K., Rickwood D. (2015). Mental health in sport: Coaches’ views of their role and efficacy in supporting young people’s mental health. Int. J. Health Prom. Ed..

[19] Brown M., Deane F.P., Vella S.A., Liddle S.K. (2017). Parents views of the role of sports coaches as mental health gatekeepers for adolescent males. Int. J. Ment. Health Promot..

[20] Hurley D., Swann C., Allen M.S., Okely A.D., Vella S.A. (2017). The role of community sports clubs in adolescent male mental health: The perspective of adolescent males’ parents. Qual. Res. Sport Exerc. Health.

[21] Vella S., Swann C. (2020). Time for mental healthcare guidelines for recreational sports: A call to action. Br. J. Sports Med..

[22] Henriksen K., Schinke R., Moesch K., McCann S., Parham W., Larsen C., Terry P. (2020). Consensus statement on improving the mental health of high performance athletes. Int. J. Sport Exerc. Psychol..

[23] Reardon C.L., Hainline B., Aron C.M., Baron D., Baum A.L., Bindra A., Budgett R., Campriani N., Castaldelli-Maia J.M., Currie A. (2019). Mental health in elite athletes: International Olympic Committee consensus statement. Br. J. Sports Med..

[24] Schinke R., Stambulova N., Si G., Moore Z. (2018). International society of sport psychology position stand: Athletes’ mental health, performance, and development. Int. J. Sport Exerc. Psychol..

[25] Atkinson M., Atkinson M. (2019). Introduction: Mental illness in sport: Sociological legacies, absences, and controversies. Sport, Mental Illness and Sociology.

[26] Roderick M., Smith A., Potrac P. (2017). The sociology of sports work, emotions and mental health: Scoping the field and future directions. Sociol. Sport J..

[27] Gale L., Ives B., Potrac P., Nelson L. (2019). Trust and distrust in community sports work: Tales from the “shop floor”. Sociol. Sport J..

[28] Ives B., Gale L., Nelson L., Potrac P., Green K., Smith A. (2016). Enacting youth sport policy: Towards a micro-political and emotional understanding of community sports coaching. The Routledge Handbook of Youth Sport.

[29] Ives B., Gale L., Potrac P., Nelson L. (2019). Uncertainty, shame and consumption: Negotiating occupational and non-work identities in community sports coaching. Sport Ed. Soc..

[30] Potrac P., Jones R., Purdy L., Nelson L., Marshall P., Potrac P., Gilbert W., Denison J. (2013). Towards an emotional understanding of coaching practice: A suggested research agenda. Routledge Handbook of Sports Coaching.

[31] Potrac P., Jones R., Gillbourne D., Nelson L. (2013). Handshakes, BBQs, and bullets: A tale of self -interest and regret in football coaching. Sports Coach. Rev..

[32] Potrac P., Smith A., Nelson L. (2017). Emotions in sport coaching: An introductory essay. Sports Coach. Rev..

[33] Roberts S., Baker M., Reeves M., Jones G., Cronin C. (2019). Lifting the veil of depression and alcoholism in sport coaching: How do we care for carers?. Qual. Res. Sport Exerc. Health.

[34] Cronin C., Armour K. (2017). ‘Being’ in the coaching world: New insights on youth performance coaching from an interpretative phenomenological approach. Sport Ed. Soc..

[35] Cronin C., Armour K. (2019). Care in Sport Coaching: Pedagogical Cases.

[36] Norman L., Rankin-Wright A. (2018). Surviving rather than thriving: Understanding the experiences of women coaches using a theory of gendered social well-being. Int. Rev. Soc. Sport.

[37] Grey-Thompson T. (2017). Duty of Care in Sport: Independent Report to Government.

[38] (2020). UK Coaching Duty to Care Toolkit and Digital Badge. https://www.ukcoaching.org/duty-to-care.

[39] Department for Work and Pensions/Department of Health (2017). Thriving at Work: The Stevenson/Farmer Review of Mental Health and Employers.

[40] Mind, Sport and Recreation Alliance and Department for Digital, Culture, Media and Sport (2019). A Guide to Implementing the Thriving at Work Standards in the Sport and Physical Activity Sector.

[41] Smith A., Jones J., Houghton L., Duffell T. (2016). A political spectator sport or policy priority? A review of sport, physical activity and public mental health policy. Int. J. Sport Policy Politics.

[42] Her Majesty’s Government (2015). Sporting Future: A New Strategy for an Active Nation.

[43] Sport England (2016). Coaching in an Active Nation: The Coaching Plan for England, 2017–2021.

[44] UK Coaching (2017). Our Strategy 2017–2021.

[45] Pike E., Atkinson M. (2019). Mental illness stigma. Sport, Mental Illness and Sociology.

[46] World Health Organization (WHO) (2014). Social Determinants of Mental Health.

